# Heart-lung crosstalk in acute respiratory distress syndrome

**DOI:** 10.3389/fphys.2024.1478514

**Published:** 2024-10-18

**Authors:** Nazareth N. Rocha, Pedro L. Silva, Denise Battaglini, Patricia R. M. Rocco

**Affiliations:** ^1^ Biomedical Institute, Department of Physiology and Pharmacology, Fluminense Federal University, Niteroi, Brazil; ^2^ Laboratory of Pulmonary Investigation, Carlos Chagas Filho Institute of Biophysics, Federal University of Rio de Janeiro, Rio de Janeiro, Brazil; ^3^ Anesthesia and Intensive Care, IRCCS Ospedale Policlinico, Genova, Italy; ^4^ Department of Surgical Sciences and Integrated Diagnostics (DISC), University of Genova, Genova, Italy

**Keywords:** acute respiratory distress syndrome, heart-lung crosstalk, mechanical ventilation, cor pulmonale, veno-venous ECMO, inflammatory mediators

## Abstract

Acute Respiratory Distress Syndrome (ARDS) is initiated by a primary insult that triggers a cascade of pathological events, including damage to lung epithelial and endothelial cells, extracellular matrix disruption, activation of immune cells, and the release of pro-inflammatory mediators. These events lead to increased alveolar-capillary barrier permeability, resulting in interstitial/alveolar edema, collapse, and subsequent hypoxia and hypercapnia. ARDS not only affects the lungs but also significantly impacts the cardiovascular system. We conducted a comprehensive literature review on heart-lung crosstalk in ARDS, focusing on the pathophysiology, effects of mechanical ventilation, hypoxemia, and hypercapnia on cardiac function, as well as ARDS secondary to cardiac arrest and cardiac surgery. Mechanical ventilation, essential for ARDS management, can increase intrathoracic pressure, decrease venous return and right ventricle preload. Moreover, acidemia and elevations in transpulmonary pressures with mechanical ventilation both increase pulmonary vascular resistance and right ventricle afterload. Cardiac dysfunction can exacerbate pulmonary edema and impair gas exchange, creating a vicious cycle, which hinders both heart and lung therapy. In conclusion, understanding the heart-lung crosstalk in ARDS is important to optimize therapeutic strategies. Future research should focus on elucidating the precise mechanisms underlying this interplay and developing targeted interventions that address both organs simultaneously.

## 1 Introduction

Acute Respiratory Distress Syndrome (ARDS) is a severe condition triggered by an initial insult (e.g., pneumonia, sepsis, trauma, or aspiration), leading to damage of lung epithelial and endothelial cells, as well as extracellular matrix (ECM), activation of immune cells, which subsequently release pro-inflammatory mediators. These pathological events increase the permeability of the alveolar-capillary barrier, resulting in interstitial/alveolar edema, lung collapse and, ultimately, hypoxemia and hypercapnia (Matthay et al., 2019; Matthay et al., 2024; Nasa et al., 2024). ARDS remains a significant challenge in critical care, not only because of its complex and multifaceted pathophysiology but also due to its high mortality rates and the long-term morbidity experienced by survivors (Gorman et al., 2022). Traditionally, ARDS management has focused primarily on lungs, with therapeutic strategies aimed at optimizing mechanical ventilation and managing fluid balance (Bellani et al., 2016; Battaglini et al., 2023a; Villar et al., 2023). However, a growing body of evidence underscores the importance of heart-lung crosstalk—a dynamic, reciprocal interaction between the cardiovascular and respiratory systems. This interplay may exacerbate pulmonary hypertension, leading to right ventricular dysfunction and acute *cor pulmonale*, which increases mortality in ARDS (Repesse and Vieillard-Baron, 2017; Vieillard-Baron et al., 2018; Puri et al., 2021; de Carvalho et al., 2023). Left ventricular (LV) dysfunction can also occur in sepsis, the leading cause of ARDS, as a result of systemic inflammation (Jones et al., 2021).

Cardiac arrest and cardiac surgery can lead to ARDS with severe implications for patient outcome (Shih et al., 2022; Sanfilippo et al., 2022). After cardiac arrest, ARDS is often triggered by aspiration, lung injury resulting from chest compressions, and ventilator-induced lung damage, combined with ischemia-reperfusion-induced inflammation. In cardiac surgery, ARDS risk is heightened by cardiopulmonary bypass, blood transfusions, and fluid shifts, all contributing to pulmonary edema. Both scenarios are linked to prolonged ICU stays, increased mortality and long-term morbidity (Rong et al., 2016).

In this review, we will examine the complex dynamics of heart-lung crosstalk in ARDS. We will explore how mechanical ventilation, fluid management, as well as hypoxemia and hypercapnia influence this interaction. Additionally, we will address the role of veno-venous extracorporeal membrane oxygenation in the management of severe ARDS. We will also discuss how cardiac arrest, and cardiac surgery may evolve to ARDS.

## 2 Pathophysiological mechanisms of heart-lung crosstalk in ARDS

ARDS is characterized by a complex interplay of pathophysiological mechanisms that affect not only the lungs but also the cardiovascular system and other vital organs (Matthay et al., 2019; Matthay et al., 2024). It can arise from both direct lung injuries, such as pneumonia and aspiration, as well as indirect injuries, including sepsis and pancreatitis (Rocco and Zin, 2005; Menezes et al., 2005). Regardless of the cause, the hallmark of ARDS is damage to the alveolar-capillary barrier, resulting in increased permeability and the development of interstitial and alveolar edema (Nasa et al., 2024).

Several inflammatory markers, including interleukin (IL)-6, IL-1β, tumor necrosis factor (TNF)-α, monocyte chemoattractant protein-1 (MCP-1), IL-8 (Cross and Matthay, 2011) and ferritin (Mehta et al., 2024) have been associated with worse patient outcomes. Moreover, biomarkers of both epithelial [surfactant protein-D (SP-D) and soluble receptor for advanced glycation end products (sRAGE)] and endothelial [angiopoietin-2 (Ang-2) and von Willebrand factor (vWF)] cell injury have been identified in ARDS (Stevens et al., 2024). However, the extent of injury to each cell type can vary significantly depending on the severity and underlying cause of the condition. This variability underscores the complexity of ARDS and highlights the need for a deeper understanding of these biomarkers to improve diagnostic accuracy and enable more personalized therapeutic approaches (Battaglini et al., 2023b). Additionally, biomarkers related to coagulopathy, such as plasminogen activator inhibitor-1 and protein C (McClintock et al., 2008), the extracellular matrix [matrix metalloproteinases (MMP-8 and MMP-9)] (Al-Husinat et al., 2024), and inflammasome activity [IL-18] (Strowig et al., 2012) have also been studied in the context of ARDS.

The systemic inflammatory response promotes the production of reactive oxygen species (ROS) and the activation of MMPs, which can further compromise both lung and heart function through oxidative stress and extracellular matrix degradation (Sipmann et al., 2018; Al-Husinat et al., 2024). ARDS is often accompanied by the release of endotoxins and damage-associated molecular patterns (DAMPs), which amplify the systemic inflammatory response promoting heart and other organ dysfunction (Huang et al., 2024). Biomarkers of cardiac stretch, such as brain natriuretic peptide (BNP) and N-terminal-pro brain-natriuretic-peptide (NT-proBNP) (Ni et al., 2020; Jayasimhan et al., 2021) as well as markers of cardiac injury (Troponin-T and Troponin-I) (Bajwa et al., 2007) are associated with higher mortality in ARDS. Severe ARDS can lead to cardiac dysfunction or injury through several mechanisms, including systemic inflammation, hypoxemia, and elevated pulmonary artery pressure, all of which increase the risk of death. Both human and animal studies have demonstrated that hypoxic pulmonary vasoconstriction can increase BNP levels (Cargill and Lipworth, 1995), and BNP is thought to have a vasorelaxant effect on the pulmonary circulation, possibly as part of the body’s compensatory response to ARDS.

The interaction between sympathoadrenal overstimulation and pro-inflammatory cytokine release creates a vicious cycle in which increased myocardial oxygen demand, driven by catecholamines, coincides with reduced contractile function due to inflammation and direct cardiac injury. This dynamic can ultimately lead to LV dysfunction, a critical factor in conditions such as septic shock. If untreated, impaired cardiac function can lead to cardiovascular collapse and multi-organ failure (Chotalia et al., 2023a; Chotalia et al., 2023b; Sinha et al., 2024). Dammassa et al. (2023) evaluated serum levels of catecholamines in patients with COVID-19-related ARDS, examining their relationships with clinical, inflammatory, and echocardiographic parameters. In their cohort of patients with moderate to severe COVID-19 ARDS, elevated norepinephrine levels were observed in individuals exhibiting right ventricular (RV) and LV systolic dysfunction. This increase in norepinephrine was also associated with higher levels of C-reactive protein (CRP) and IL-6, correlating with increased mortality rates ().

**FIGURE 1:**
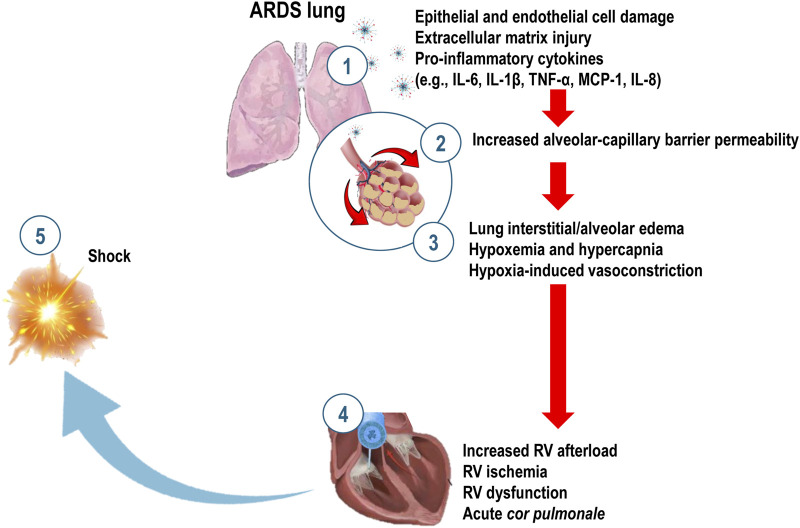
Mechanisms of inflammation and heart-lung interaction in Acute Respiratory Distress Syndrome (ARDS). Pathogen exposure or cellular damage leads to injury of lung epithelial and endothelial cells, as well as the extracellular matrix. This damage activates alveolar macrophages and immune cells, initiating a cascade of inflammatory responses involving tumor necrosis factor-alpha (TNF-α), interleukin-1β (IL-1β), IL-6, monocyte chemoattractant protein-1 (MCP-1), and IL-8, and the recruitment of neutrophils **(1)**. These inflammatory processes further damage the alveolar-capillary barrier **(2)**, resulting in lung interstitial and alveolar edema, atelectasis, hypoxemia, and hypercapnia **(3)**. Hypoxia-induced vasoconstriction **(3)** increases right ventricular (RV) afterload, leading to RV ischemia, dysfunction, acute *cor pulmonale* **(4)** and, ultimately shock **(5)**.

In healthy lungs, hypoxic pulmonary vasoconstriction (HPV) redistributes blood flow (Q’) to areas with better ventilation (V’), optimizing ventilation-perfusion (V’/Q’) matching. However, in ARDS, this redistribution is impaired, resulting in areas of high perfusion but low ventilation (intrapulmonary shunting) and areas of high ventilation but low perfusion (dead space ventilation). Poor perfusion in well-ventilated alveoli can be due to thrombosis or edema-induced vascular blockage, which increases pulmonary vascular resistance (PVR) and leads to severe hypoxemia (Ryan et al., 2014) (Figure 1). Hypoxia also hinders alveolar fluid clearance (Huppert and Matthay, 2017) and decreases sodium (Na+) transport activity in alveolar-epithelial type II cells, a process that can be reversed by reoxygenation (Planès et al., 1996; Vivona et al., 2001).

Zapol and Snider were the first to document that elevated PVR is a common finding in patients with severe respiratory failure (Zapol and Snider, 1977). The development of pulmonary arterial hypertension (PAH) and right ventricular dysfunction can be associated with the following interconnected mechanisms: 1) interstitial/alveolar edema leads to hypoxemia, compresses extrinsic vessels and puts stress on the pulmonary vasculature, thus increasing PVR (Price et al., 2012; Pradhan et al., 2020; Cusack et al., 2023); 2) endothelial dysfunction in ARDS promotes the recruitment of inflammatory cells, such as neutrophils and macrophages, into the pulmonary circulation, leading to the release of more pro-inflammatory mediators, contributing to vascular damage and remodeling; 3) inflammation and oxidative stress can impair the endothelial nitric oxide synthase (eNOS) pathway, reducing nitric oxide production, resulting in vasoconstriction and increased PVR; 4) endothelial damage reduces the production of prostacyclin, an important vasodilator produced by endothelial cells. This reduction promotes vascular constriction, further contributing to increased PVR; and 5) lung overdistention can elevate PVR by increasing transpulmonary pressure on the vessels surrounding the alveoli (Whittenberger et al., 1960).

**FIGURE 2:**
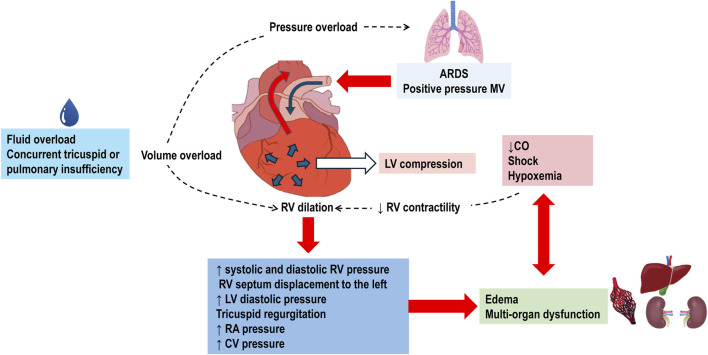
Pathophysiology of Right Ventricular Failure in Acute Respiratory Distress Syndrome (ARDS). The use of positive pressure mechanical ventilation (MV) imposes pressure overload on the right ventricle (RV). This overload is further exacerbated by fluid overload and concurrent tricuspid or pulmonary valve insufficiencies. In response to the increased pressure, the RV dilates, leading to elevated systolic and diastolic RV pressures. The dilated RV pushes the interventricular septum towards the left ventricle (LV). This displacement increases LV pressure, causes tricuspid regurgitation, and raises right atrial (RA) and central venous (CV) pressures. The resultant systemic congestion contributes to organ dysfunction. Compression of the LV by the dilated RV diminishes cardiac output (CO). Coupled with shock and hypoxemia, this reduction in CO exacerbates systemic congestion and the risk of multi-organ failure. Decreased RV contractility due to shock further worsens RV dilation, perpetuating a cycle of right ventricular failure.

Pulmonary hypertension increases the afterload on the RV, resulting in acute *cor pulmonale* (Pinsky, 2016; Parker et al., 2023). Normally, the RV ejects blood into the pulmonary circulation, which is a low-resistance and high-compliance system with minimal isovolumetric contraction pressure and insignificant isovolumetric relaxation, acting as a passive conduit. The systolic function of RV is highly sensitive to increases in PVR and has limited adaptation reserves, making it susceptible to dysfunction and dilation. This dilation of the RV can compress the LV, contributing to circulatory failure. When the RV is overloaded during systole, its contraction is prolonged, causing it to exert pressure even after the LV has completed its contraction. This results in higher pressure in the RV cavity compared to the LV cavity for a brief period, leading to paradoxical septal motion. This motion decreases LV diastolic compliance and stroke volume. Reduced cardiac output further diminishes coronary perfusion, exacerbating RV ischemia and deteriorating RV contractility, which can progress to cardiogenic shock and death (Pinsky, 2016; Peck and Hibbert, 2019) (Figure 2).

Studies evaluating RV dysfunction in ARDS have focused on three echocardiographic parameters: estimated pulmonary artery (PA) systolic pressure, RV dimensions and function, and septal dyskinesis. These parameters are used to classify RV dysfunction into three categories: 1) no dysfunction (estimated PA systolic pressure ≤40 mmHg with no RV impairment), 2) moderate dysfunction (estimated PA systolic pressure >40 mmHg with RV dilation but without septal dyskinesis), and 3) severe dysfunction or *cor pulmonale* (RV dilation, decreased systolic function, and septal dyskinesis) (Vieillard-Baron et al., 2001). In ARDS, acute *cor pulmonale* is present in 21%–50% of patients mechanically ventilated and is associated with increased mortality (Ganeriwal et al., 2023)

Sub-phenotypes are defined as distinct subgroups of ARDS based on a shared, measurable characteristics, which can be reliably discriminated from other sub-phenotypes. In this line, four cardiovascular sub-phenotypes have been identified in ARDS patients (Chotalia et al., 2023a): 1) preserved RV: normal biventricular function; 2) RV dilation but preserved function and/or cardiac output; 3) RV failure with RV dilation and dysfunction with low cardiac output; and 4) hyperdynamic: high cardiac output and left ventricular failure (Paonessa et al., 2015). The highest mortality rate (78%) was observed in group 3, characterized by RV failure. Group 4 is linked to high systemic inflammation (Chotalia et al., 2023b). In a previous analysis focusing on cardiovascular sub-phenotypes in COVID-19 patients (Chotalia et al., 2022), three sub-phenotypes based on right ventricular function were described: 1) preserved RV function or normal RV; 2) RV dysfunction with dilation but preserved function; and 3) RV failure with dilation and severely impaired RV function. In short, the recognition of these phenotypes is important not only for predicting outcomes but also for developing more specific therapeutic strategies.

Bacterial (Suffredini et al., 1989; Shenoy et al., 2018) and viral (Zheng et al., 2020) infections are common causes of sepsis-induced ARDS and can also lead to cardiomyopathy (Zainab et al., 2023). The inflammatory response triggered by these infections plays a central role in the pathophysiology, contributing to both direct myocardial injury and systemic inflammation, which ultimately results in RV failure or even biventricular dysfunction (Zainab et al., 2023). In addition to inflammation, sepsis activates coagulation pathways and leads to endothelial dysfunction, impairing microvascular blood flow and tissue hypoxia (Russell, 2006). In the context of COVID-19, the exaggerated inflammatory response, often referred to as a “cytokine storm,” has been closely associated with severe ARDS and cardiac involvement, particularly LV dysfunction (Moore and June 2020).

## 3 Impact of mechanical ventilation on heart-lung interactions

The interaction between the heart and lungs is influenced by their shared anatomical location within the thoracic cavity and direct vascular connections. As the heart is located within the thorax, fluctuations in intrathoracic pressure (ITP) during the respiratory cycle significantly influence the pressures applied to the heart. ITP acts as the external pressure surrounding the heart, and right atrial pressure (Pra), in relation to RV filling, is most accurately described as Pra minus ITP—referred to as transmural pressure. Similarly, LV ejection pressure can be estimated as arterial pressure minus ITP. Both transmural Pra and LV pressures vary dynamically with changes in ITP during respiration. Pra serves as a key determinant of venous return, opposing mean systemic filling pressure (Pmsf). Throughout the ventilatory cycle, variations in Pra directly affect venous flow rates. During inspiration, as pleural pressure (Ppl) decreases, Pra reduced. This decrease in Pra enhances venous return, leading to an increase in RV end-diastolic volume and subsequently boosting RV stroke volume in the next cardiac cycle. Conversely, during expiration, as Ppl becomes less negative, Pra rises to its end-expiratory value, resulting in a reduction in venous return (Grübler et al., 2017; Mahmood and Pinsky, 2018).

Mechanical ventilation, a cornerstone in the management of ARDS, affects heart-lung interactions, with both therapeutic and adverse effects (Mahmood and Pinsky, 2018). While mechanical ventilation is crucial for maintaining adequate oxygenation in ARDS patients, its impact on cardiovascular function is complex and requires careful consideration (Jardin and Vieillard-Baron, 2003; Cortes-Puentes et al., 2018; Sklar et al., 2019; Sklar and Munshi, 2022). During passive mechanical ventilation, both alveolar pressure and ITP remain positive throughout the respiratory cycle, reaching their lowest point at end-expiration. The rise in ITP is transmitted to the right atrium, reducing the pressure gradient between the venous system and the right atrium (Marini et al., 1981), thus decreasing venous return (Morgan et al., 1966). This decrease in preload can compromise cardiac output, particularly affecting the ability of RV to effectively pump blood into the pulmonary circulation (Paonessa, et al., 2015). The resultant decrease in cardiac output can exacerbate systemic hypotension and contribute to organ dysfunction, complicating the management of critically ill patients (Magder, 2016; Bouferrache and Vieillard-Baron, 2011; Corp et al., 2021). An increase in intra-abdominal pressure during insufflation can raise mean systemic filling pressure by redistributing blood from the unstressed to the stressed volume, though this effect is less pronounced in cases of volume depletion. Additionally, baroreceptor-driven sympathetic activation may further increase mean systemic pressure during insufflation, helping to minimize the reduction in venous return (Jozwiak and Teboul, 2024).

**FIGURE 3:**
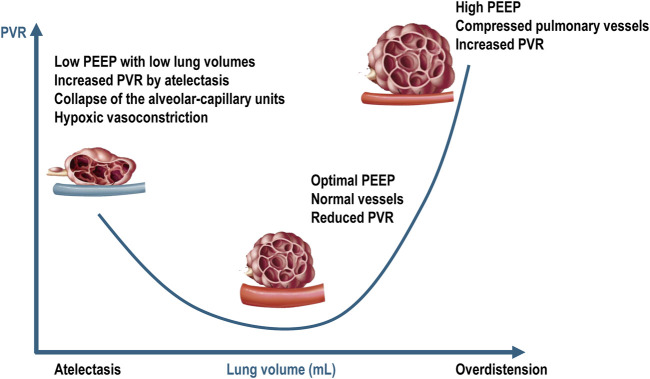
Relationship between pulmonary vascular resistances and intra-alveolar and extra-alveolar vessels. PEEP = positive end-expiratory pressure; PVR = pulmonary vascular resistance. Blue vessel represents deoxygenated blood. Adapted from West JB, Luks AM. *West’s Respiratory Physiology: The Essentials*. Philadelphia, PA: Wolters Kluwer Health, 2015; p. 92; and Alviar CL et al. *Positive Pressure Ventilation in the Cardiac Intensive Care Unit*. *Journal of the American College of Cardiology*, 2018; 72 (13):1532–1553.

From a serial component viewpoint, the pulmonary circulation may be divided in extra-alveolar vessels and intra-alveolar vessels. As lung volume increases from residual volume to total lung capacity, the alveolar capillary vessels become increasingly compressed due to lung overdistension, leading to increased vascular resistance. In contrast, the resistance in the extra alveolar vessels decreases as they become less tortuous and more dilated (Whittenberger et al., 1960). This creates a “U-shaped” relationship between PVR and lung volume, with the opposing effects of alveolar and extra-alveolar blood vessels being normally balanced at functional residual capacity. In patients with ARDS undergoing mechanical ventilation, these effects are difficult to predict because lung collapse and overdistension occur simultaneously and to varying degrees. Several factors exacerbate the compression of pulmonary capillaries in ARDS, including interstitial edema, alveolar collapse, smooth muscle constriction due to hypoxia and hypercapnia, vascular remodeling, and the direct impact of positive intrathoracic pressure (Marini et al., 2003). The capacity to accommodate blood flow is reduced in ARDS due to inflammation, consolidation, compressive vascular collapse, and microthrombosis (Tomashefski et al., 1983). Additionally, reduced capillary reserve leads to elevated mean vascular pressure. High lung volumes can exacerbate lung overdistension, increasing RV afterload and potentially leading to pulmonary hypertension (Jardin and Vieillard-Baron, 2007; Walkey et al., 2017; McGuigan et al., 2024) (Figure 3).

During insufflation, increased transpulmonary pressure can shift blood from the pulmonary venous circulation to the left atrium, enhancing LV filling (Vieillard-Baron et al., 2003). However, reduced venous return and impaired RV ejection during insufflation can decrease LV preload during the following expiration, due to the long pulmonary transit time. Although RV stroke volume often decreases during insufflation, significant rightward septal shift is unlikely, as studies have shown no change in RV end-diastolic volume during this phase (Vieillard-Baron et al., 2003). Left ventricular afterload reduced by the increase in ITP, which is transmitted to the LV and thoracic aorta, lowering transmural aortic pressure and facilitating LV ejection. This interaction between respiratory and circulatory systems can improve stroke volume, particularly in patients with LV dysfunction.

In ARDS, changes in ITP due to mechanical ventilation are usually too small to significantly alter hemodynamics because of low lung compliance and protective low tidal volume strategies. While fluctuations in transpulmonary pressure still influence RV afterload, the use of low tidal volumes helps minimize these variations during the ventilatory cycle (Grasselli et al., 2023).

To evaluate the effects of tidal ventilation on the heart, changes in esophageal pressure (P_ES_) can be measured to estimate the right atrial pressure. This estimation allows for more precise adjustments to ventilation strategies, aiming to minimize adverse cardiovascular effects. However, routine measurement of P_ES_ remains challenging (Talmor et al., 2006).

The use of PEEP in mechanical ventilation significantly influences cardiovascular dynamics in ARDS patients (Fougères et al., 2010; Joseph et al., 2024). On the one hand, PEEP is beneficial for recruiting collapsed alveoli, the end-expiratory lung volume increases toward FRC, thus reducing heterogeneous regional strain and shunt, as well as improving oxygenation (Dantzker et al., 1980), which in turn reduce hypoxic vasoconstriction, thereby lowering PVR. On the other hand, it can increase intrathoracic pressure, potentially leading to lung overdistension (biophysical trauma) and PVR, reduce RV preload, and worsen RV dysfunction, particularly in severe ARDS (Ganeriwal et al., 2023). In such cases, the balance between optimizing ventilation and preserving cardiac function becomes especially challenging (Zainab et al., 2023; Battaglini et al., 2024b). Moreover, since ARDS lungs contain both recruitable and normal alveoli, the effects of PEEP on PVR depend on the balance between alveolar recruitment and overdistension (Cappio Borlino et al., 2024). This variability explains why ARDS patients show differing RV responses to PEEP.

PEEP also increase ITP, which raises right atrial pressure and may reduce venous return. However, in ARDS patients, this reduction is mitigated due to airway pressure transmission caused by decreased lung compliance. Additionally, PEEP may elevate mean systemic pressure by increasing intra-abdominal pressure and triggering adaptive mechanisms (Verzilli et al., 2010), such as sympathetic activation. Positive pressure ventilation may also activate the renin-angiotensin-aldosterone system, leading to splanchnic venoconstriction, which shifts blood into the central circulation (Gelman, 2008). These mechanisms can increase venous resistance, potentially complicating hemodynamic management.

Traditionally, oxygenation is targeted, when setting PEEP, with the fraction of inspired oxygen (FiO2) adjusted to maintain arterial oxygen levels within a specific range. This approach, however, assumes that hypoxemia is primarily due to alveolar collapse and a consequent reduction in lung compliance. However, in patients with ARDS, this assumption is often inadequate because hypoxemia can occur even when lung compliance is minimally impaired. When PEEP fails to recruit functional lung units and does not improve pulmonary compliance, it may result in lung overdistension and excessive energy transfer to the pulmonary-parenchymal matrix, increasing the risk of ventilator-induced lung injury and adverse hemodynamic effects. Therefore, setting PEEP to target optimal lung compliance can mitigate these risks, though this strategy may have its own drawbacks (Cove et al., 2022; Grieco et al., 2022). The level of PEEP should be individualized as its hemodynamic effects depend on how it influences both RV afterload and preload.

Recent data from small-animal models with previously healthy lungs have demonstrated the adverse effects of abruptly releasing high levels of PEEP (Katira et al., 2018). This phenomenon is primarily attributed to cardiovascular compromise due to the initial “surge” of translocating fluid volumes from peripheral to central vascular compartments. A recent study evaluated the effects of abrupt *versus* gradual PEEP release, combined with standard or high fluid volumes, on lung and heart in experimental ARDS. Abrupt PEEP compared to gradual PEEP decrease associated with high fluid volumes worsen lung damage and increased the expression of pro-inflammatory markers. However, gradual PEEP reduction with standard fluid volumes helped preserve lung epithelial integrity and maintain better overall hemodynamics (Rocha et al., 2021).

The distinction between positive pressure mechanical ventilation (passive) and spontaneous breathing (active) is important in the management of ARDS, as it significantly influences heart-lung interactions and cardiovascular function. In fully passive mechanical ventilation, the ventilator controls the entire respiratory cycle, leading to uniform increases in ITP during inspiration (Luecke and Pelosi, 2005). This rise in ITP decreases venous return to the right heart by reducing the pressure gradient between systemic veins and the right atrium, impairing RV filling. The elevated ITP reduces LV afterload by compressing thoracic vasculature, making it easier for the LV to eject blood. In patients with ARDS, where lung function is already compromised and pulmonary vascular resistance is elevated, these effects can further strain the cardiovascular system, complicating the management of critical patients (Luecke and Pelosi, 2005). In contrast, during active or spontaneous breathing, as seen in modes like pressure support ventilation, the respiratory muscles, particularly the diaphragm, contract and generate negative ITP during inspiration (Takata and Robotham, 1992). This negative ITP increases venous return (Roos et al., 1961), potentially enhancing cardiac output, compared to passive ventilation (Guyton et al., 1957). However, during expiration, the positive pressure effects of mechanical ventilation persist, creating a dynamic interplay between negative pressure inspiration and positive pressure expiration (Marini, 2011). In ARDS patients, spontaneous breathing efforts can either improve or complicate ventilation and cardiovascular performance, depending on the severity of lung injury and the ventilator settings.

Chest wall compliance significantly influences the transmission of ventilator pressures to the heart, particularly in ARDS patients with obesity, where respiratory system, lung, and chest wall compliance are often reduced (Magder et al., 2021). In such cases, the stiff chest wall in obese patients causes much of the ventilator-applied pressure to be directed towards distending the chest wall rather than the lungs. Managing ARDS in obese patients requires clinicians to carefully balance ventilator settings to optimize lung recruitment while minimizing excessive pressure transmission to the heart (Silva et al., 2022). Strategies such as appropriate PEEP levels, prone positioning, and esophageal pressure monitoring can help mitigate the negative hemodynamic impacts (Bonatti et al., 2019). Higher PEEP levels in obese ARDS patients reduce detrimental heart–lung interactions (Magder et al., 2021). The primary benefit arises from improving respiratory system compliance, which allows for a lower driving pressure to ventilate the lungs, consequently reducing the compromise of RV function.

### 3.1 Electrical impedance tomography

Electrical impedance tomography (EIT) is a non-invasive imaging modality that offers real-time, bedside monitoring of regional lung ventilation and perfusion, making it particularly valuable in managing ARDS (Frerichs et al., 2017; Bachmann et al., 2018). EIT is increasingly employed to guide PEEP titration, optimizing lung recruitment while reducing the risk of ventilator-induced lung injury (VILI). Although PEEP can improve oxygenation by preventing alveolar collapse and ensuring even ventilation distribution, excessive levels may elevate intrathoracic pressure, compromising venous return and cardiac output (Costa et al., 2009; Zhao et al., 2019). EIT can detect small pulsatility signals that correlate with stroke volume. While its accuracy may be insufficient for comprehensive hemodynamic monitoring in critically ill patients, the precision of this pulsatility signal could suffice for calibrating relative V’/Q’ mismatch (Leali et al., 2024). Integrating lung aeration and perfusion monitoring remains a significant challenge for EIT devices. Future advancements may enable non-invasive stroke volume variation estimation, thereby enhancing heart-lung interaction monitoring. Additionally, synchronizing EIT with other respiratory signals, such as esophageal pressure, could provide clinicians with simultaneous, complementary data that has been previously inaccessible. Although EIT is predominantly used in hypoxemic respiratory failure requiring invasive mechanical ventilation, its application could extend to non-intubated patients and perioperative monitoring during abdominal or thoracic surgery (Franchineau et al., 2024).

### 3.2 Prone positioning

Prone positioning improves oxygenation (70%–80% of cases) in severe ARDS patients. This improvement is primarily due to better V’/Q’ matching, which occurs as edema redistributes within the injured lungs (Jozwiak et al., 2013). When a patient is placed in the prone position, the previously dependent dorsal lung regions, which become non-dependent, reinflate, while the ventral regions, now dependent, may collapse. However, since lung perfusion remains relatively unchanged, the overall V’/Q’ matching improves, allowing newly recruited pulmonary units to engage in more effective gas exchange (Jozwiak et al., 2013). Prone positioning not only improves oxygenation but may also reduce RV afterload (Jozwiak et al., 2016). Additionally, prone positioning can increase intra-abdominal pressure (IAP), which might boost venous return and cardiac preload. However, this effect depends on the level of IAP, as excessive pressure may collapse the inferior vena cava. If cardiac preload increases, the impact on cardiac output will depend on the patient’s preload reserve. Finally, prone positioning can also increase LV afterload due to the elevated IAP. The overall effect on cardiac output will vary depending on the interplay of these mechanisms (Jozwiak et al., 2013; Jozwiak et al., 2016; Boesing et al., 2024).

### 3.3 Diaphragm neurostimulation

Diaphragm neurostimulation is an emerging technology that aims to mitigate the adverse effects of mechanical ventilation. Rohrs et al. investigated temporary transvenous diaphragm neurostimulation (TTDN), which synchronizes diaphragm contractions with ventilator breaths, generating negative pressure during inspiration. This method, called negative-pressure-assisted ventilation, showed promise in preclinical studies by reducing atelectasis, improving oxygenation (PaO2/FiO2), and decreasing lung injury (Rohrs et al., 2021; 2022). Additionally, TTDN can enhance venous return and cardiac output by creating a transdiaphragmatic pressure gradient, particularly in preload-dependent conditions (Masmoudi et al., 2017). Diaphragm-protective ventilation through neurostimulation offers a promising future in mechanical ventilation management.

## 4 Hypoxemia, hypercapnia, and cardiac function

The interaction between hypoxemia, hypercapnia, and cardiac function is a critical aspect of ARDS pathophysiology (Repessé and Vieillard-Baron, 2017). Hypoxemia impairs myocardial function by reducing oxygen delivery to the heart, exacerbating myocardial ischemia and potentially worsening heart failure. As a result, decreased oxygen levels lead to reduced cardiac contractility, lower cardiac output, and increased PVR and RV strain, further compromising cardiovascular function (Thomas et al., 2022).

Hypercapnia induces respiratory acidosis, which diminishes myocardial contractility and exacerbates cardiac stress in ARDS. Additionally, hypercapnia can impair cardiac electrical conduction, increasing the risk of arrhythmias and destabilizing cardiac function. Respiratory acidosis also alters vascular tone, leading to systemic vasodilation and hypotension, which complicates the maintenance of adequate perfusion in ARDS patients (Repessé and Vieillard-Baron, 2017; Morales-Quinteros et al., 2019; Ganeriwal et al., 2023).

Hypercapnia affects multiple organ systems (Curley et al., 2010). While it enhances alveolar ventilation and corrects V’/Q’ mismatch, it also reduces myocardial contractility. In response, sympathetic stimulation can result in tachycardia to maintain adequate cardiac output, provided the patient has enough cardiovascular reserve (Curley et al., 2010). However, hypercapnia can worsen pulmonary vasoconstriction, raising PVR and leading to mean pulmonary arterial pressure (Wang et al., 2008). This can worsen *cor pulmonale*, where the RV is strained due to high pulmonary pressures (Rodarte and Hyatt, 1973; Viitanen et al., 1990). Additionally, hypercapnic acidosis impairs immune function by reducing neutrophil migration to infection sites and inhibiting phagocytic activity (Coakley et al., 2002). In humans, hypoxemia combined with severe hypercapnia has been associated with diminished renal function, which may explain the increased need for hemodialysis (Kilburn and Dowell, 1971).

Mekontso Dessap et al. (2009) used transesophageal echocardiography (TEE) in patients with severe ARDS and found that hypercapnic acidosis induced by low-tidal volume ventilation and increasing PEEP at constant plateau pressure directly impaired RV function, independent of PEEP effects.

Furthermore, hypoxemia and hypercapnia can amplify systemic inflammation, exacerbating endothelial cell dysfunction and increasing vascular permeability, which can adversely affect cardiac function and overall hemodynamic stability. The combined effects of hypoxemia, hypercapnia, and systemic inflammation can create a vicious cycle of worsening respiratory and cardiac dysfunction, complicating the clinical course of ARDS and increasing the risk of adverse outcomes (Harjola et al., 2016; Swenson and Swenson, 2021).

## 5 Veno-venous ECMO and heart-lung crosstalk

The Berlin definition of ARDS is crucial for categorizing the severity of the syndrome, which guides ventilator management and therapeutic decisions. Moderate ARDS is identified by a PaO2/FiO2 ratio of 101–200 mmHg, reflecting shunt fractions between 20% and 60%. Severe ARDS, however, is characterized by higher shunt fractions, increased lung weight, and a pronounced hyperinflammatory response (Battaglini et al., 2023a)

VV-ECMO has become a pivotal intervention for severe ARDS, particularly when conventional mechanical ventilation fails to ensure adequate oxygenation and ventilation (Pappalardo and Montisci, 2018). This technique provides extracorporeal gas-exchange support, allowing severely damaged lungs to rest and recover while maintaining systemic oxygen delivery and carbon dioxide removal. VV-ECMO directly addresses gas-exchange deficits, effectively managing severe hypoxemia and hypercapnia, thus improving tissue oxygenation and mitigating systemic effects of respiratory failure. Additionally, VV-ECMO can reverse pathophysiological mechanisms associated with ARDS, such as hypoxemia-induced pulmonary vasoconstriction and *cor pulmonale*, thereby enhancing cardiac function and systemic hemodynamics (Combes et al., 2018; Combes et al., 2024).

The interaction between VV-ECMO and cardiac function is critical in managing severe ARDS. While VV-ECMO can reduce RV afterload by improving pulmonary oxygenation and decreasing hypoxic pulmonary vasoconstriction, it may also lower preload, potentially reducing cardiac output if not properly managed (Combes et al., 2024).

Despite its benefits, VV-ECMO presents challenges, including complex anticoagulation management, which requires balancing clot prevention in the circuit with minimizing bleeding risks. Additionally, VV-ECMO is associated with potential complications such as infections, hemolysis, and organ dysfunction. Careful patient selection, involving a thorough assessment of underlying conditions and comorbidities, is essential to optimize outcomes and minimize adverse effects (Giraud et al., 2021; Fior et al., 2022; Bagate et al., 2024; Battaglini et al., 2024a).

## 6 ARDS secondary to cardiac arrest and cardiac surgery

In patients experiencing in-hospital cardiac arrest, ARDS develops within 3 days in up to 72% of cases, leading to increased mortality rates and poorer neurological outcomes compared to those who do not develop ARDS (Shih et al., 2022). The onset of ARDS following cardiac arrest can be attributed to several factors: aspiration, lung contusion from chest compressions, ventilator-induced lung injury, and ischemia-reperfusion injury (Langeland et al., 2022).

Diagnosing ARDS after cardiac surgery is particularly challenging due to the underlying cardiac dysfunction that complicates the clinical presentation (Rong et al., 2016). Significant risk factors for ARDS in this context include prolonged extracorporeal circulation and multiple blood transfusions, with incidence rates ranging from 0.4% to 8.1% (Hu et al., 2023). Prolonged extracorporeal circulation, often necessary in complex cardiac procedures, imposes considerable physiological stress that contributes to lung injury. Additionally, multiple transfusions, commonly required during such surgeries, can introduce inflammatory mediators that exacerbate lung damage. Patients undergoing these procedures are also at an increased risk of myocardial ischemia. The stress and systemic inflammatory response from extended extracorporeal circulation, along with the hemodynamic effects of multiple transfusions, can compromise myocardial oxygen supply and lead to ischemic events. The interaction between myocardial ischemia and ARDS is particularly concerning because compromised cardiac function can further impair oxygen delivery, exacerbate respiratory distress, and complicate patient management and recovery.

To mitigate the risk of postoperative ARDS, strategies should include optimizing mechanical ventilation, restricting blood product transfusions to maintain a hemoglobin target of 7 g/dL, minimizing hemodilution, avoiding fluid overload, and considering the hemodynamic impact of mechanical ventilation on RV function (Sanfilippo et al., 2022).

## 7 Therapeutic pharmacological strategies to improve heart-lung crosstalk

Pharmacological strategies to enhance heart-lung interaction in ARDS focus on optimizing both pulmonary and cardiovascular function. One approach involves the use of pulmonary vasodilators, such as inhaled nitric oxide (iNO) or prostacyclins, which can selectively reduce pulmonary hypertension and decrease RV afterload without causing systemic hypotension (Moloney and Evans, 2003; Adhikari et al., 2014). By improving pulmonary blood flow and oxygenation, these agents can help reduce the strain on the RV and enhance overall cardiac function. However, the benefits of these therapies must be weighed against potential risks, such as rebound pulmonary hypertension or impaired ventilation-perfusion matching (Gerlach et al., 2003; Adhikari et al., 2014). In addition to pulmonary vasodilators, the management of systemic inflammation and oxidative stress is crucial in ARDS. Corticosteroids may be used to modulate the inflammatory response, potentially improving lung function and reducing fluid accumulation in the lungs, which can benefit cardiac performance by decreasing the burden on the heart (Chaudhuri et al., 2024). In acute experimental RV failure, the use of norepinephrine and epinephrine increase RV contractility (Apitz et al., 2012). The use of vasopressors might be necessary to support cardiac output in ARDS patients with significant cardiovascular compromise, ensuring adequate tissue perfusion while minimizing adverse effects on the lungs (Monnet et al., 2023). The careful selection and timing of these pharmacological interventions are essential for achieving optimal heart-lung interaction and improving outcomes in patients with ARDS.

## 8 Conclusion

Recognizing the heart-lung crosstalk in ARDS highlights the importance of a comprehensive approach to patient management. Integrating cardiac and respiratory assessments, optimizing ventilatory settings, managing fluids effectively, and exploring new pharmacological therapies can significantly enhance ARDS treatment. Ongoing research into the mechanisms of heart-lung interactions and the development of advanced therapeutic strategies will be crucial for improving patient outcomes.
